# Mechanism of Gene Amplification via Yeast Autonomously Replicating Sequences

**DOI:** 10.1155/2015/387367

**Published:** 2015-01-20

**Authors:** Shelly Sehgal, Sanjana Kaul, M. K. Dhar

**Affiliations:** School of Biotechnology, University of Jammu, Jammu 180006, India

## Abstract

The present investigation was aimed at understanding the molecular mechanism of gene amplification. Interplay of fragile sites in promoting gene amplification was also elucidated. The amplification promoting sequences were chosen from the* Saccharomyces cerevisiae* ARS, 5S rRNA regions of* Plantago ovata *and* P. lagopus*, proposed sites of replication pausing at *Ste20* gene locus of* S. cerevisiae,* and the bend DNA sequences within fragile site FRA11A in humans. The gene amplification assays showed that plasmid bearing APS from yeast and human beings led to enhanced protein concentration as compared to the wild type. Both the* in silico* and* in vitro* analyses were pointed out at the strong bending potential of these APS. In addition, high mitotic stability and presence of TTTT repeats and SAR amongst these sequences encourage gene amplification. Phylogenetic analysis of* S. cerevisiae* ARS was also conducted. The combinatorial power of different aspects of APS analyzed in the present investigation was harnessed to reach a consensus about the factors which stimulate gene expression, in presence of these sequences. It was concluded that the mechanism of gene amplification was that AT rich tracts present in fragile sites of yeast serve as binding sites for MAR/SAR and DNA unwinding elements. The DNA protein interactions necessary for ORC activation are facilitated by DNA bending. These specific bindings at ORC promote repeated rounds of DNA replication leading to gene amplification.

## 1. Introduction

Gene amplification represents a cellular process characterized by the production of multiple copies of a particular gene or genes, thereby leading to their enhanced expression. In some organisms it is an integral part of the normal developmental process [1] or is closely associated with abnormal processes, such as malignancies [2], increased drug resistance [3], and mutations [4]. Dhar et al. [5] reported origin of a complete chromosome due to amplification of rRNA sequences in* Plantago*. Significant effort in this field was the discovery of* cis*-acting genetic element* aps* (amplification promoting sequence) from the nontranscribed spacer region of tobacco ribosomal DNA (rDNA), which reportedly increased the level of expression of recombinant proteins [6].

In yeast genome, gene amplification is closely associated with specific DNA sequences known as autonomously replicating sequences or ARS. These sequences are identified by their unique ability of high frequency transformation and stable plasmid maintenance [7]. ARS are short DNA sequences of few hundred base pairs which support maintenance of plasmid in growing yeast cells. Some of these ARS elements are known to behave as origins of replication [8, 9]. ARS elements are spread over sixteen chromosomes of yeast on an average once every 30–40 kb. Apart from the extrachromosomal origin function, many but not all ARS elements also function as replication origins in their original chromosomal context [10, 11]. A strong ARS has been estimated to yield 50,000 transformants/*μ*g of DNA, while the weakest ARS yields < 100 transformants/*μ*g of DNA [12]. Based on the time taken for replication initiation, the origins in yeast have been classified into early and late replicating [13]. Dhar et al. [14] reported a comprehensive compilation on the distribution of the ARS within the genus* Saccharomyces*. The present study offers the physical aspects governing replication efficiency and mechanism of gene amplification* via* ARS elements. A thorough understanding of these elements may aid in their better utilization for the development of specialized yeast vectors for scientific and commercial applications in genetic engineering.

## 2. Materials and Methods

### 2.1. Isolation of Amplification Promoting Sequences

Five different sources were used for the isolation of amplification promoting sequences (APS). (I) For the* in silico* analysis, all the ARS located on the sixteen* S. cerevisiae* chromosomes were considered (~740). Only some of the ARS elements proposed to behave as CEOs (compromised early origins) were taken into consideration for experimental purposes. These were ARS310, ARS315, ARS606, ARS806, ARS1305, ARS1426, and ARS1512; (II) nontranscribed spacer (NTS) region of 5S ribosomal DNA of* P. ovata* (366 bp); (III) full 5S rDNA region (363 bp; NTS region is 242 bp) was chosen from* P. lagopus* to check its efficacy as amplification promoting sequence; (IV) sites of replication fork impediment at* Ste20* gene located on chromosome VIII of* S. cerevisiae* [15] and (V) regions prone to DNA bending within fragile site FRA11A. FRA11A maps to 11q13.3 region within the 11q13 locus of the human chromosome. The DNA sequence of the FRA11A retrieved from NCBI was analyzed in depth for identifying the regions of maximum curvature and DNA bending using* bend.it* and TWIST-FLEX programs. The 11 Mb sequence of FRA11A was analysed using several bioinformatics tools. Although there were many regions with curvature > 14, two regions were found with curvature > 16. These regions are FSI—11445795–11455795 and FSII—11451795–11452795 which were selected for further analysis (Table 1).

### 2.2. PCR Amplification

**Figure 1:**
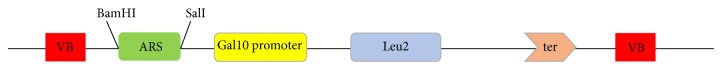
Cloning of amplification promoting sequences. The vector used for cloning APS sequences was the yeast episomal plasmid YEp51.

Genomic DNA was isolated from yeast strains (Table 2), plants, and resected normal and tumor tissues using standard protocols [16–18]. Total RNA was removed from the DNA samples by treatment with RNaseA at a concentration of 10 *μ*g/mL. Specific primers were designed for amplification of APS using IDT (Integrated DNA Technologies) online software. Oligonucleotide primers for each APS were designed and were flanked with the* Bam*H1 and* Sal*1 restriction sites at the 5′ and the 3′ ends. For* Bam*H1 “GGATCC” sequence was used and for* Sal*1 “GTCGAC” was used. Trinucleotides “GCA” or “CAG” were also used as support at the 5′ end as done previously by Venkateswarlu et al. [19]. The sequences of all the primers used in this study are listed in Table 3. The APS sequences were PCR amplified using specific primers. Desired fragments were eluted from the gel using GenAxy DNA mini elution kit. YEp51G (yeast episomal plasmid) was used for vector cassette construction (Figure 1). It is a shuttle plasmid bearing leucine and ampicillin as the markers. The vector was transformed in* S. cerevisiae* using LiAc method (Table 4). Confirmation of cloning was done by colony PCR as reported by Akada et al. [20] and restriction digestion. Direct sequencing reaction confirmed the presence of desired fragments in the clones.

### 2.3. Gene Amplification Assays

Crude yeast cell extract was prepared. Bradford assay was done for determining concentration of solubilized protein [21]. Mitotic stability and plasmid loss rate assays were performed as described by Dani and Zakian [22] with slight modifications. For curvature analysis of APS native 10% polyacrylamide gel electrophoresis was carried out to confirm the bending intensities of the fragments showing noticeable bendability during* in silico* analysis. The protocol was adopted from Bechert et al. [23]. The migration of the marker fragments was determined and calibration curves were plotted (logarithm of the base pairs versus distance migrated). The calibration graph for the length determination of the APS DNA incorporating fragments was performed individually for each gel.

### 2.4. *In Silico* Analysis

After assessing the amplification potential of the APS by gene amplification assays, the sequences were further analysed using several bioinformatics tools (WEB-THERMODYN, TREP,* model.it*,* bend.it*, SDSC, TwistFlex, EMBOSS, and RNAfold). This analysis was essentially carried out to investigate the role of sequential behaviour of the APS in promoting gene amplification. SGD and oriDB databases were used for collation of information. Also, an effort was made to identify the presence and location of known replication enhancer (RE) sequences and matrix attachment regions (MAR) within these APS [24]. A 189 bp region has crucial role for ARS activity and scaffold binding being reported earlier by Amati et al., 1990 [25]. Presence of this sequence in* S. cerevisiae* ARS was checked using BLAST. Geneious Pro (5) software was used for the phylogenetic analysis of* S. cerevisiae* ARS elements using neighbour joining method (Table 5).

## 3. Results

**Figure 2:**
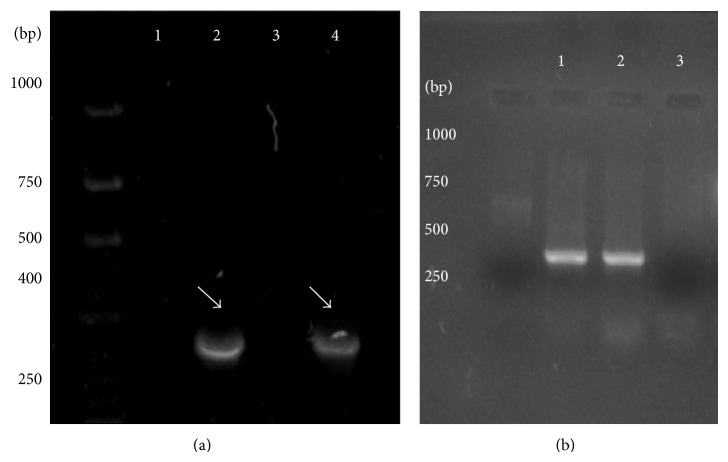
PCR amplification of the APS from (a)* P. ovata* and (b)* P. lagopus*. (a) The migrating bands for the 5S rRNA sequence PCR amplified from the genomic DNA of* P. ovata*. Lanes 2 and 4 show the 350 bp band corresponding to the PCR amplified APS. Lanes 1 and 3 served as control. In (b) lane 3 served as control and 350 bp bands in lanes 1 and 2 correspond to the PCR amplified 5S rRNA sequence. M is the 100 bp DNA ladder.

**Figure 3:**
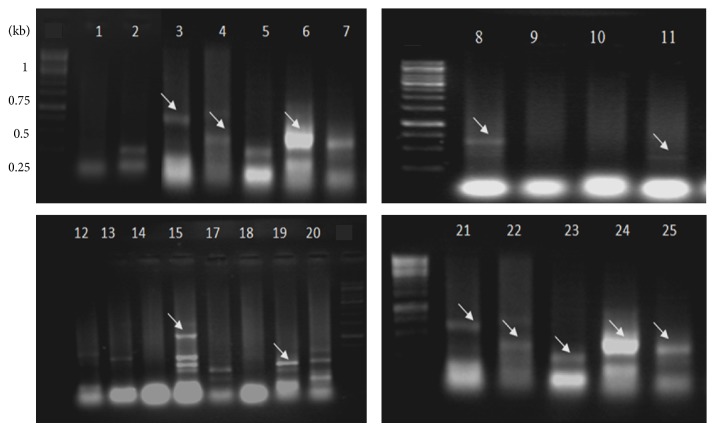
PCR amplification of the ARS from* S. cerevisiae*. Lane 1 is the control and in lane 3 428 bp band corresponds to the mol. wt. of the ARS315. Lane 4 represents the expected band of the 367 bp corresponding to ARS606. Bright band of 381 bp representing ARS1512 (lane 6), 281 bp band corresponding to ARS1305 (lane 8), 328 bp band corresponding to ARS1426 (lane 15). Lanes 21–25 represent the reamplified ARS and lane 21 represents ARS315; lane 22, ARS606; lane 23, ARS1305; lane 24, ARS1512; and lane 25, ARS1426. Some primer combinations used did not yield desired products (lanes 2, 5, 7, 9, 10, 12, 13, 14, 17, 18, and 20). M is the 1 kb molecular wt marker.

**Figure 4:**
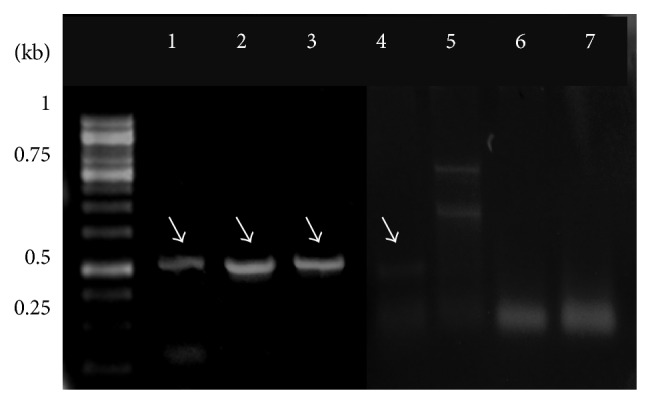
PCR amplification of the fragile sites located at the* Ste-20* gene locus present on chromosome VIII of* S. cerevisiae*. Lane 1: band of the mol. wt. 613 bp corresponding to Ste-YF1, lane 2: 529 bp band representing Ste-YF2, lane 3: 531 bp band corresponding to Ste-YF3, lanes 6 and 7: control, and lane 4: band of mol. wt. 487 bp representing Ste-YF4. M is the 100 bp molecular wt marker and L is the 1 kb DNA ladder.

**Figure 5:**
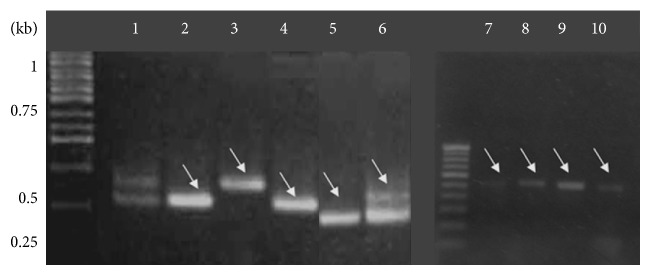
PCR amplification of the fragile site regions present within the FRA11F fragile site locus on chromosome 11q13 in humans. Lane 1 has the positive control for PCR amplification. Lane 2 and lane 4: PCR amplicon of 546 bp corresponding to FRAI. Lane 3: band of mol. wt. 689 bp corresponding to FRA II. Lanes 7–10: reamplified FRAI sequence. M is the 1 kb DNA ladder.

Genomic DNA was successfully isolated from* S. cerevisiae*,* P. ovata*,* P. lagopus*, and resected tissue samples. Amplification products within 320–350 bp size range corresponding to 5S rRNA of* P. ovata* and* P. lagopus* were observed (Figure 2). The amplification products of different ARS were observed as differentially migrating bands. Amplicon corresponding to ARS315 was of the expected size of ~428 bp, while that of ARS606 was ~367 bp. A bright band of size ~381 bp was observed representing ARS1512. PCR amplification products of ARS1305 and ARS1426 were comparatively of lower molecular weight, that is, ~281 bp and ~328 bp, respectively (Figure 3). PCR product of the expected molecular weight ~613 bp was observed corresponding to Ste-YF1, while the PCR amplicon (~529 bp) was corresponding to Ste-YF2, and ~531 bp amplification product was obtained for Ste-YF3. PCR product representing Ste-YF4 was also successfully amplified (~487 bp) (Figure 4). PCR amplicon of ~546 bp was observed corresponding to FRAI, while amplification product of ~689 bp represented FRAII (Figure 5).

Increase in the activity was analysed spectrophotometrically from the crude cell lysates. Each sample was analysed in three biological replicates to confirm the values obtained. Protein activity increased significantly in the cells having the plasmid as compared to the wild type cells without the plasmid. Maximum activity was observed in the pYEp51GA-6 followed by pYEp51GA-13, while wild type pYEp51G showed the minimum activity. Except pYEp51GA-1 and pYEp51GA-2 which showed insignificant increase in expression of the GUK1 gene, the remaining constructs showed an increase in the protein activity (Table 6). The transformation efficiency and the mitotic stability of the cells were calculated as shown in Table 7. It was observed that Yep51GA-3, Yep51GA-5, Yep51GA-8, YEp51GA-12, and YEp51GA-13 were more stable as compared to the other transformants over the generations. High plasmid loss indicative of the low mitotic stability was observed in the pYEp51GA-9, pYEp51GA-7, and the wild type.

**Figure 6:**
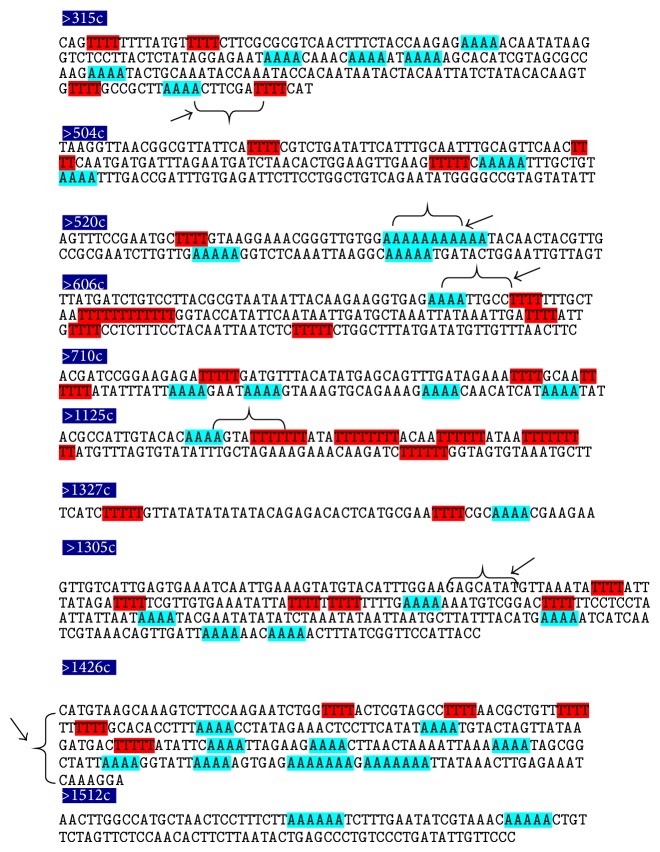
Nucleotide repeat pattern search in* S. cerevisiae* ARS elements. Sequences of the highest curvature regions of the* S. cerevisiae* ARS elements. The tetranucleotide repeats of “T” are shown in red ink, while those of “A” are shown in green ink.

**Figure 7:**
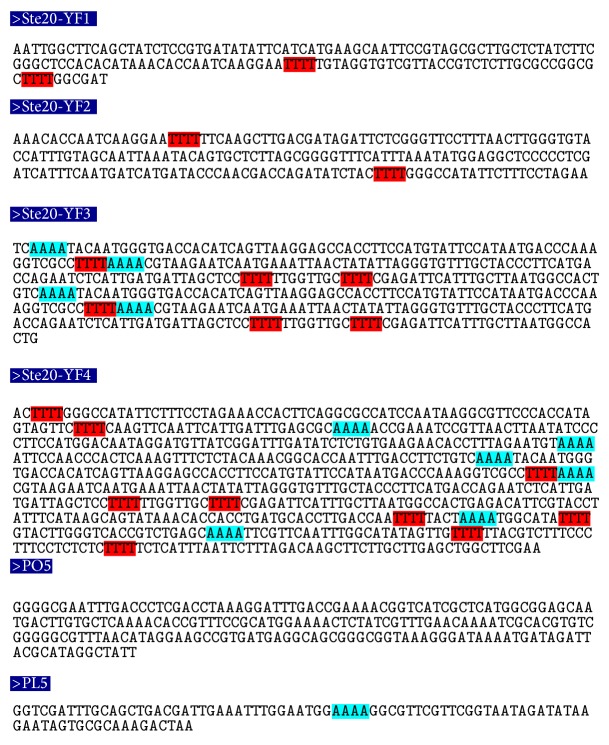
Nucleotide repeat pattern search in APS from other eukaryotes. Pattern search in the highest curvature regions of the APS isolated from plants, yeast, and human beings. The tetranucleotide repeats of “T” are shown in red ink, while those of “A” are shown in green ink.

In most of the ARS elements, it was found that the tetranucleotide stretches of “A” followed by “TTTT” were present in the regions depicting highest curvature (Figure 6). Long stretches of “A” were also located in the bend regions of ARS506 and ARS1426, while extended “T” stretches were also detected (e.g., ARS606 and ARS1125). Interesting observation was that the ARS showing the maximum bending tendencies in experimental (Table 8) and theoretical analysis (ARS315, ARS606, ARS1305, and ARS1426) had A4 repeats followed by T4 repeats (highlighted by arrows in Figure 7). Also, there were ample trinucleotide sequences like TTA and ATA known to have high curvature values in the regions of ARS elements under study. The present observations regarding DNA bending at APS fragments indicate the strong possibility of the presence of replication enhancer sequences within them which promote DNA bending and hence may lead to gene amplification (Table 9). Out of the six RE sequences used for the present analysis, RE2, RE4, and RE6 were completely absent in any of the ARS, while RE3 and RE5 were generously present in many ARS. Also, presence of RE1 was observed in ARS1127, while Ste20-YF3 and FSII showed the presence of RE3. Moreover, ARS315, ARS606, ARS1305, ARS1426, and ARS1512 showed maximum homology with 189 bp sequence. FRAI showed lesser similarity as compared to FRAII with this sequence. A general observation was that all the selected APS had high similarity with this sequence, which points towards the pathway of formation of secondary structures by these APS during gene amplification.

**Figure 8:**
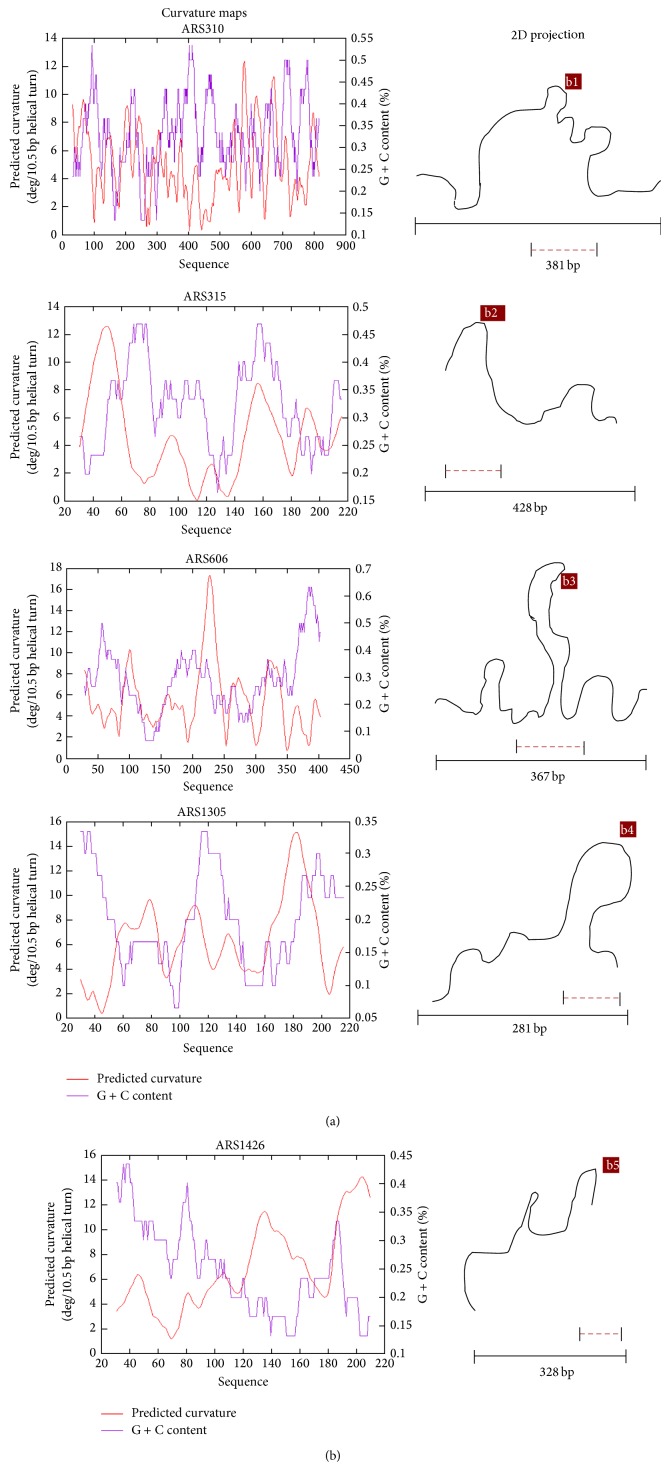
Curvature maps and 2D projection of the amplification promoting sequences. Regions of maximum bending are shown in ARS310 (b1), ARS315 (b2), ARS606 (b3), ARS1305 (b4), and ARS1426 (b5).

**Figure 9:**
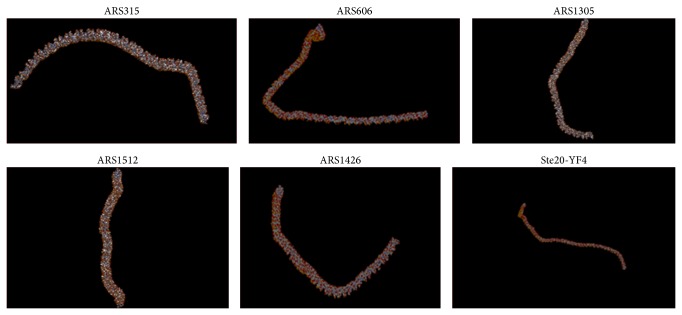
Three-dimensional view of the amplification promoting sequences. The figures show the 3D structure of the APS depicting the inbuilt curvature in the DNA sequences. These were made using the* model.it* software.

Curvature propensity and bendability values of the selected APS sequences are listed in Table 10. The magnitude of the predicted curvature propensity was within the range calculated for experimentally tested curved motifs (>1 and <22.5) for all the APS except those obtained from plants. Based on these results, the 2D projections depicting exact regions of DNA bending amongst the ARS are shown in Figure 8. Further, local bending of selected APS was predicted by static-geometry models as well using* model.it* DNA analysis tool. 3D models of the bent DNA were created from the sequence data and gave the output as a PDB file (Figure 9).

**Figure 10:**
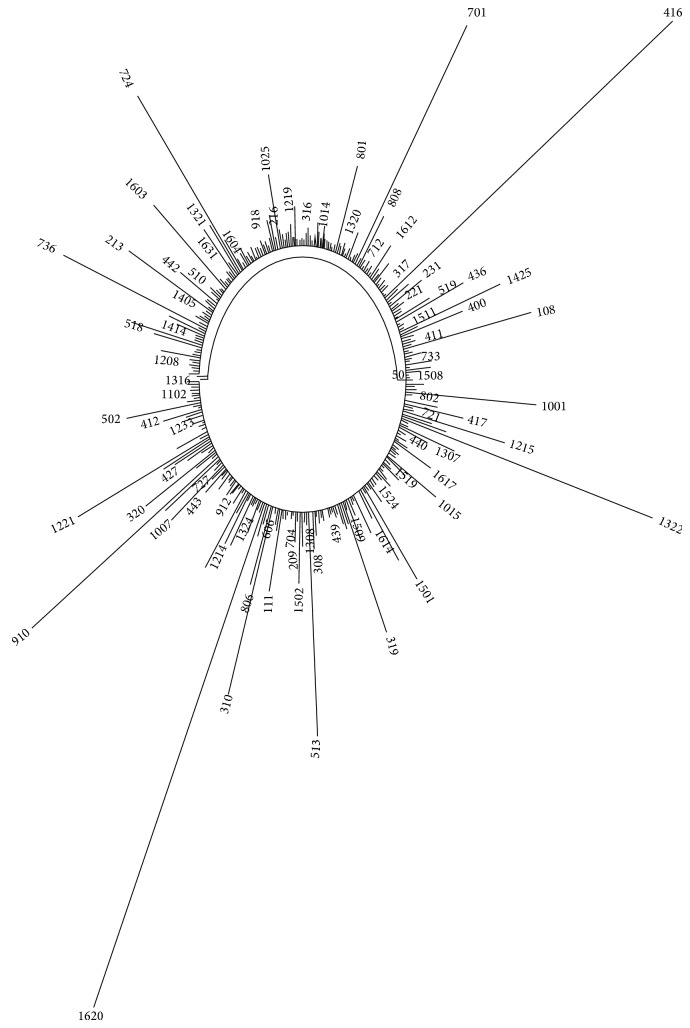
Phylogenetic analysis of the* S. cerevisiae* ARS. Phylogenetic tree constructed by using the neighbourhood joining method.

The analysis of hereditary molecular differences amongst these ARS elements was carried out in order to gain information about their evolutionary relationships. The phylogram generated in this manner had three main branches (Figure 10), where ARS1316 and ARS1507 appeared as outliers with bootstrap value of 100, while the rest of the ARS were clustered in the same branch. In phylogenetic analysis, branch length is a measure of the amount of divergence between two nodes in a tree. The ARS1620 showed the maximum branch length, followed by ARS701 and ARS416. Apart from these, ARS502, ARS513, ARS1221, ARS910, ARS310, ARS319, ARS1302, ARS1001, and ARS108 also showed longer branch lengths as compared to the rest. ARS located on chromosome V showed longer branch lengths as compared to others (ARS518, ARS503, ARS515, ARS519, ARS513, ARS504, and ARS502). A noteworthy observation was that ARS located on the same chromosome were located very close to each other in the dendrogram, for example, ARS of chromosome VI (ARS1618, 1607, 1608, 1633, 1603, 1626, 1629, 1631, and 1604), chromosome IV (ARS409, 428, 432, 418, 442, 406, 403, 453, 400, 421, and 411), chromosome XII (ARS1208, 1213, 1200-1, 1200-2, and 1220), and chromosome XIV (ARS1414, 1409, and 1405). We found significant closeness between the ARS of chromosome IV and ARS of chromosomes XII, V, and VII (ARS434-1219, 435-1212, 436-1234-400-421, 450-1202-26, 446-1218, and 452-1230). Also, ARS of chromosomes III and XIII (ARS308-1308, 310-1323, 307-1333, 320-1312, 309-1307, and 304-1317) and chromosomes XIV and XV (ARS1420-1518, 1423-1510, 1415-1521, and 1406-1526) showed close relatedness. Phylogenetic analysis also pointed out that the ARS containing intact ACS sequence were also evolutionary proximal. Adjacent location of the very similar sequences further confirms the accuracy of the tree (ARS1200-1 and 1200-2).

To correlate the evolutionary trends of the ARS with their physical properties, a combined analysis of the eight parameters (i.e., free energy values (Δ*G*), *T*_REP_ values, origin efficiency, SIDD, flanking elements, colocalization of a possible fragile site, curvature, and GC content) of each of these chromosomal ARS was conducted (see Supp I in Supplementary Material available online at http://dx.doi.org/10.1155/2015/387367).

## 4. Discussion

Identification of the key players of gene amplification process and their inducing factors is a challenge to a number of biological processes in both prokaryotes and eukaryotes [2]. The aim of the present research was to understand the mechanism of gene amplification* via* fragile sites. Therefore, ARS elements known to behave as CEOs (compromised early origins) which are known to cause fragility in yeast were chiefly considered [26]. Slight differences in significant and conserved functional sequence motifs within ARS can modulate their ORC binding affinity and origin activity [14]. Although the basis of origin inefficiency of CEO ARS elements is not clear, it is possible that the chromatin conformation of these ARS is such that it results in weaker binding of the ARS to* trans*-acting factors crucial for origin activation. This has been proposed as a possible explanation for the fact that only one origin, that is, ARS607, is used in >85% of cells in chromosome VI [27].

Gene amplification assays performed during the present investigation showed that plasmid bearing amplification promoting sequences from yeast and human beings had significantly enhanced protein activity. It has been documented that the effect of APS is likely due to a combination of (i) its structural features such as its 80% A+T content, (ii) repetitive ARS core elements, (iii) DNA bending, and (iv) SAR-related (scaffold attachment region) sequences [6]. All of these factors play a crucial role in formation of protein-DNA complexes that initiate and regulate nearby gene amplification and transcription. Synergistic effect of these factors may explain a significant increase in gene expression associated with the vector cassette bearing ARS1426 and FRAII. The bioinformatics analysis was essentially carried out to investigate the role of sequential behaviour of the APS in promoting gene amplification. The exact regions harbouring the bend DNA, RE sequences, and MARs amongst the chosen APS were also successfully located.

Out of all the APS chosen for the present investigation, selected 189 bp SAR was found in the APS elements derived from yeast and humans. This specific SAR has been reported in* Drosophila*, where 189 bp region from the 5′ SAR element of* fushi tarazu* (ftz) gene was found to be crucial for ARS activity and scaffold binding [25]. Similar studies have also shown that 40% of the 58* Drosophila* SARs tested function as ARS elements in yeast [28]. Nuclear scaffold interacts with genomic DNA at these specific sites (SARs) and forms the basis of the DNA loops. This indicates that the AT-rich genomic DNA present in these APS elements remains specifically attached to residual nuclear structures. A correlation between the replication enhancer sequences and SARs was evident from earlier studies which confirm that several scaffold binding sites coincide or map very near to enhancer elements [29]. Further, strong affinity of these regions with DNA stem-loops or cruciforms has been reportedly seen frequently during eukaryotic replication [30]. For example, the 14-3-3 proteins present in yeast, plants, amphibians, and invertebrates, located within the nucleus, are involved in eukaryotic DNA replication via binding to the cruciform DNA that forms transiently at replication origins [31]. Also, deletion of the cruciform binding domain of the protein at the* ori* site leads either to reduction or failure in replication in budding yeast [32]. This analysis indicates a positive correlation between scaffold binding affinity and ARS activity as homologs of 14-3-3 proteins in* S. cerevisiae*, Bmh1p and Bmh2p, have cruciform DNA-binding activity and associate* in vivo* with ARS307 [30].

DNA bending is known to play a key role in the formation of nucleoprotein structures, as well as in the specific interaction of proteins with their DNA sites. Many studies have suggested that DNA bending was necessary for origin activity [33]. Such regions of DNA bending have been reported in yeast ORC, promoter regions of prokaryotes,* GAL1-10* and* GAL80* regulatory genes of yeast, regions of nucleosome formation, and recombination sites using electrophoretic and circular permutation analyses [34, 35]. Both* in silico* and* in vitro* analyses of DNA bending pointed out strong bending potential of some of the APS, like FRAII and Ste20-YF3, apart from the* S. cerevisiae* CEO ARS-ARS315, ARS606, ARS1305, ARS1426, and ARS1512. The results of this analysis are in line with the studies of Hagerman [36], wherein it was proposed that direct repeats of the sequence GA4T4C gave rise to bending, while GT4A4C did not. Our observations are also in coherence with the preliminary model for ARS elements given by Eckahl and Anderson [37]. AT stretches have been associated with increased instability and gene amplification events in yeast [38].

Much surprisingly, plant APS (PO5 and PL5) could not efficiently trigger gene amplification. Minimum DNA bending tendencies, high plasmid loss indicative of the low mitotic stability, detection of only the tetranucleotide “A” repeats, and absence of TTTT repeats and SAR related sequences may explicate this behaviour. Even though DNA bending was not observed in the plant APS chosen for this study, bent DNA regions have been seen in mutant proteins of transcriptionally active OccR-octopine complexes in plant tumors [39]; plant MADS-box proteins [40] and rRNA promoter upstream sequences in* Arabidopsis thaliana* [41].

To correlate the evolutionary trends of the ARS with their physical properties, a combined analysis of the eight parameters (i.e., free energy values (Δ*G*), *T*_REP_ values, origin efficiency, SIDD, flanking elements, colocalization of a possible fragile site, curvature, and GC content) of each of these chromosomal ARS was conducted (Supp I). This was compared with their closely placed members in the phylogenetic tree. A thorough comparison of the properties of the members of each cluster revealed interesting results. It was observed that, in addition to the closeness in the phylogenetic tree, ARS1316 and 1507 also share similarity with respect to their physical properties. They behave as confirmed origins of replication and have low curvature (9-10), low TREP, and low G+C values. ARS1620 which showed the longest branch length had high SIDD value, no flanking elements, and low curvature (approximately 7). These properties were similar to the properties of ARS701 which had the second longest branch length. ARS elements of chromosome IV were found to be located close to one another and approximately all the members showed similar properties; for example, ARS409, 432, and 418 showed replication efficiency, fragile site colocalization, high curvature, and Δ*G* and *T*_REP_ values (curvatures 14–16 were noteworthy). Similarly, ARS518, 503, 515, 519, and 513 showed very similar properties, while ARS of chromosomes IV and XII showed very similar properties. ARS308–1308 were more closely placed in the phylogram and this close relatedness was well reflected in their physical properties (high Δ*G*, low curvature, presence of LTR, and fragile sequences).

**Figure 11:**
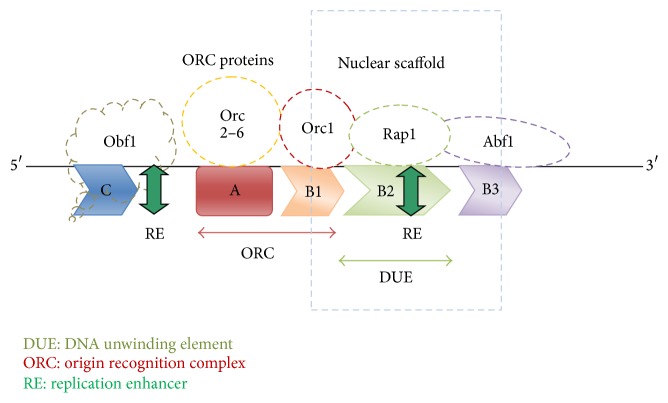
Location of SAR and RE sequences at the* S. cerevisiae* ARS. Three domains of the* S. cerevisiae* ARS, that is, A, B, and C, are shown. Also the approximate locations of the nuclear scaffold attachment regions and replication enhancer sequences based on our analysis are shown. Different types of proteins that bind to different domains of ARS are also shown in the figure.

The combinatorial power of all the aspects of amplification promoting sequences analyzed in the present investigation was harnessed to reach a consensus on the factors which stimulate gene expression in presence of these sequences. A thorough comparison of the properties of the members of each cluster revealed interesting results. Approximately, all the closely placed members showed similar properties, for example, replication efficiency, fragile site colocalization, high curvature, and Δ*G* and *T*_REP_ values. It is worth mentioning here that the analysis of APS has shown that the replication enhancer sequences and SARs are also localized in their high curvature regions. Higher DNA bendability evident at the regions prone to gene amplification can hence be attributed partly to the presence of the tetranucleotide repeats of A and T, SAR like sequences, and replication enhancer sequence in this region. The protein binding sites in the* S. cerevisiae* ARS may play a crucial role in stimulation of origin activity. It has been reported that DNA-binding site and the OBF1 protein are involved in the regulation of the activation of nuclear origins of replication in* S. cerevisiae*; that is, OBF1 DNA-binding site is an enhancer of DNA replication [42]. The location of the SAR in Figure 11 is also confirmed by an earlier study which reported that the highly AT-rich region forms the 5′ boundary of the functional gene regulatory domains [25].

**Figure 12:**
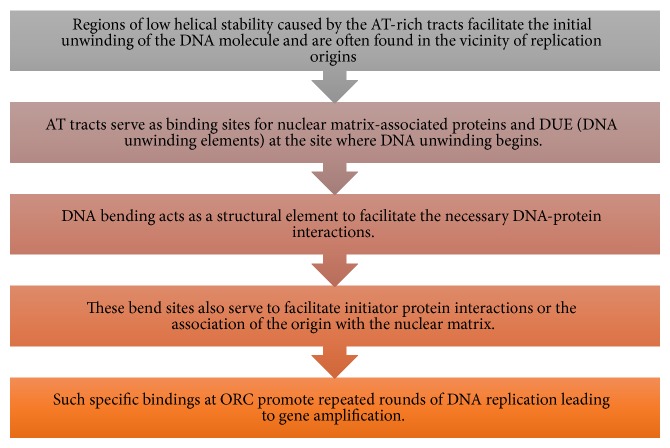
Mechanism of gene amplification based on combined analysis of thermodynamic factors. The steps involved in the origin and progression of gene amplification in* S. cerevisiae* ARS.

The collated results of the analysed features of the dissected* S. cerevisiae* ARS are represented diagrammatically (Figure 11). Based on the experiments carried out in the present study, a mechanism of gene amplification was hypothesized (Figure 12). In nutshell, the mechanism of gene amplification in ARS (autonomously replicating sequence) of* S. cerevisiae* was that AT-rich tracts present in fragile sites of yeast serve as binding sites for MAR/SAR and DNA unwinding elements (Figure 12). The DNA protein interactions necessary for ORC activation are facilitated by DNA bending. These specific bindings at ORC promote repeated rounds of DNA replication leading to gene amplification. The combinatorial power of all the aspects of amplification promoting sequences analyzed in the present investigation paved a way to reach a consensus on the factors which stimulate gene expression, in presence of these sequences.

## Supplementary Material

Combined analysis: To correlate the evolutionary trends of the ARS with their physical properties, a combined analysis of the eight parameters (i.e., free energy values (∆*G*), *T*_REP_ values, origin efficiency, SIDD, flanking elements, co-localization of a possible fragile site, curvature and GC content) of each of these chromosomal ARS was conducted. This was compared with their closely placed members in the phylogenetic tree. A thorough comparison of the properties of the members of each cluster revealed interesting results. It was observed that in addition to the closeness in the phylogenetic tree, ARS 1316 and 1507 share similarity with respect to their physical properties too. They behave as confirmed origins of replication, have low curvature (9-10), low TREP and low G+C values. ARS 1620 which showed the longest branch length had high SIDD value, no flanking elements and low curvature (approximately 7). These properties were similar to the properties of ARS 701 which had the second longest branch length. ARS elements of chromosome IV were found to be located close to one another and approximately all the members showed similar properties, for example, ARS 409, 432, 418 showed replication efficiency, fragile site colocalization, high curvature, ∆*G* and *T*_REP_ values (curvature 14–16 was noteworthy). Similarly, ARS 518, 503, 515, 519, 513 showed very similar properties. Also, ARS of chromosome IV and XII showed very similar properties, while ARS 308-1308 were more closely placed in the phylogram and this close relatedness was well reflected in their physical properties also (high ∆*G*, low curvature, presence of LTR and fragile sequences).

## Figures and Tables

**Table 1:** Flexibility analysis of FRA11A sequence.

S. number	Base pairs	Curvature	TWIST FLEX flexibility index		
			Peaks	Unified peaks	Cluster of peaks
FS1	11055795–11165795	16–18	3	1	1
FS2	11165795–11265795	14	2	2	—
FS3	11265795–11365795	15	1	1	—
FS4	11365795–11465795	16	1	1	—
FS5	11465795–11565795	15	1	1	—
FS6	11565795–11665795	15	2	2	—

**Table 2:** Strains and plasmids used for gene amplification assays.

S. number	Strains/plasmid	Description
1	MTCC 762	“*α*” CUP1 SUC2 gal2 mal mel

2	MTCC 3089	“a” ura3 trp1 lys2 his3 leu2 PFY1

3	YEp51	Amp[R], *E. coli*-yeast shuttle vector, GAL10 promoter

4	YEp51G	Amp[R], *E. coli*-yeast shuttle vector, GAL10 promoter, GUK1 gene

**Table 3:** Primers used for the PCR amplification of APS used in this study.

APS	Primer	Primer sequence (5′-3′)	Product size (bp)	*T*_*m*_ (°C)
ARS315	315 F	GGGATACGACCGGTCCTGAGAAA	428	56
ARS315	315 R	CCGCAAAGATGTGCGGGAAATTCT	428	56
ARS606	606 F	GGTCTTCTTGATAATTCTGTGGGCGCT	367	57
ARS606	606 R	GGTACCATATGAAACTCCAGCAGC	367	57
ARS1305	1305 F	TGGCCATTGCGTTGTCATTGAGTG	281	59
ARS1305	1305 R	GGGTGGAGTAATGGGTTTCACAAAGG	281	59
ARS1426	1426 F	ACCGTAGCCGTAACGTTTAATGCC	328	57
ARS1426	1426 R	CGAGGAGATCCTCTTCTCTTTGCT	328	57
ARS1512	1512 F	TCCAATGGCTTAGTAGCTGCCAGA	381	59
ARS1512	1512 R	TGACAACAAGGAGGACAGCGAACA	381	59
PO5	PO5 F	GGGTGCGATCATACAGCGT	320	49
PO5	PO5 R	GGGTGCCAACACTAGGACTTC	320	49
PL5	PL5 F	GAGTTCTGATGGGATCCGGTG	350	51
PL5	PL5 R	CGCTTGGGCTAGAGCAGTAC	350	51
Ste20-YF1	YF1F	AAATGTCCAAATATACACGATAAG	613	54
Ste20-YF1	YF2R	AGAACTACTATGGTGGGAACG	613	54
Ste20-YF2	YF2F	TGTGTTGTCAGCAGAATGATCCGGG	529	53
Ste20-YF2	YF2R	CGAGGTAGCAAGCAACCCAAACTT	529	53
Ste20-YF3	YF3F	ATGAAGCAATTCCGTAGCGCTTGC	531	54
Ste20-YF3	YF3R	AGGCGACCTTTGGGTCATTATGGA	531	54
Ste20-YF4	YF4F	TGCTCGTTGTCGTTATCGTCGTCA	427	57
Ste20-YF4	YF4R	AGAACTACTATGGTGGGAACGCCT	427	57
FRAI	FRAI F	ACTTCAATCCGACAATGAATGGG	546	56
FRAI	FRAI R	ACAGAGCGAAATTCTATATC	546	56
FRAII	FRAII F	GGCGATACCGGTGGCAATTCTT	689	55
FRAII	FRAII R	CATGGGTATCCGTTGTCGTT	689	55

**Table 4:** APS from different sources used for the present study.

S. number	Clone	Origin
1	YEp51GA-1	5S rRNA sequence from the genomic DNA of *P. ovata *
2	YEp51GA-2	5S rRNA sequence from the genomic DNA of *Plantago lagopus *
3	YEp51GA-3	Autonomous replicating sequences from *S. cerevisiae *ARS315
4	YEp51GA-4	Autonomous replicating sequences from *S. cerevisiae* ARS606
5	YEp51GA-5	Autonomous replicating sequences from *S. cerevisiae *ARS1305
6	YEp51GA-6	Autonomous replicating sequences from *S. cerevisiae *ARS1426
7	YEp51GA-7	Autonomous replicating sequences from *S. cerevisiae *ARS1512
8	YEp51GA-8	Proposed fragile sites located at the *Ste-20* gene (YF1)
9	YEp51GA-9	Proposed fragile sites located at the *Ste-20* gene (YF2)
10	YEp51GA-10	Proposed fragile sites located at the *Ste-20* gene (YF3)
11	YEp51GA-11	Proposed fragile sites located at the *Ste-20* gene (YF4)
12	YEp51GA-12	Fragile sequences from the FRA11F (FRAI)
13	YEp51GA-13	Fragile sequences from the FRA11F (FRAII)

**Table 5:** Software/online tools used in the present study.

Database/software	Address
SGD	http://www.yeastgenome.org/
oriDB	http://www.oridb.org/index.php
WEBTHERMODYN	http://www.gsa.buffalo.edu/dna/dk/WEBTHERMODYN/
TREP	http://www.oridb.org/index.php
bend.it	http://www.icgeb.trieste.it/dna/
TwistFlex	http://bioinfo.md.huji.ac.il/marg/Flexstab/
model.it	http://www.icgeb.trieste.it/dna/
RasMol	http://biology.kenyon.edu/BMB/RASWIMAN.HTM
BL2SEQ	http://workbench.sdsc.edu/ (http://seqtool.sdsc.edu/CGI/BW.cgi)
CLUSTALW	http://www.ebi.ac.uk/Tools/msa/clustalw2/
banana	http://services.cbib.u-bordeaux2.fr/pise/banana.html
EMBOSS	http://www.ebi.ac.uk/Tools/emboss/
RNAfold	http://rna.tbi.univie.ac.at/cgi-bin/RNAfold.cgi
GeneiousPro	Software package
KaleidaGraph 3.0	http://www.synergy.com/kg.htm

**Table 6:** GUK1 activities in yeast strains (Sp activity *µ*mol/min per mg of protein).

S. number	Plasmid	Concentration of protein (mg/mL)
1	pYEp51G	0.75
2	pYEp51GA-1	0.78
3	pYEp51GA-2	0.81
4	pYEp51GA-3	1.28
5	pYEp51GA-4	1.07
6	pYEp51GA-5	1.13
7	pYEp51GA-6	1.17
8	pYEp51GA-7	0.94
9	pYEp51GA-8	1.18
10	pYEp51GA-9	0.99
11	pYEp51GA-10	1.11
12	pYEp51GA-11	0.98
13	pYEp51GA-12	1.21
14	pYEp51GA-13	1.89

**Table 7:** Assessment of mitotic stability of the constructs.

Transforming plasmid	Colony size	Doubling time in selective media (hrs)	Avg. number of plasmids/cell	% cells with plasmids	% loss of plasmid per generation
pYEp51G wt	**+**	6.0 ± 0.5	2 ± 1	3 ± 2	9 ± 2
pYEpGA-3	++	2.2 ± 0.3	33 ± 2	58	4 ± 1
pYEpGA-4	++	2.5 ± 0.5	30 ± 2	47	3 ± 2
pYEpGA-5	+	4.3 ± 0.4	19 ± 2	34	5 ± 2
pYEpGA-6	++	2.0 ± 0.3	2 ± 1	47	4 ± 1
pYEpGA-7	++	3.3 ± 0.2	6 ± 2	40	8 ± 1
pYEpGA-8	+	2.7 ± 0.2	19 ± 1	49	4 ± 2
pYEpGA-9	+++	3.0 ± 0.2	9 ± 2	42	7 ± 2
pYEpGA-12	++	2.0 ± 0.2	34 ± 2	60	4 ± 1
pYEpGA-13	++	2.2 ± 0.3	33 ± 3	62	3 ± 2

**Table 8:** Calculation of the *k* factor from the PAGE gel.

Construct	*k* factor (0.5X TBE) BB 10°C	EtBr 24°C	End-to-end distance (nm)
ARS315	1.29	1.20	72
ARS606	1.06	1.03	92
ARS806	1.11	1.06	87
ARS1305	1.14	1.06	80
ARS1426	1.12	1.05	86
FSI	1.02	1.04	69
FSII	1.01	1.04	98

**Table 9:** Presence of RE sequences in APS of *S. cerevisiae* and FRA11A.

RE sequences	CEO ARS/FRA sequences with RE
CGGAGGGGCCCTAGAGGGCCCTAGAG GGCCCCCCAAAAACCCCCAAAAACCC CCC	ARS1127

TCTCTAAAAAATATATAAAAA	—

AAAAAAAAAAAAAAAAAAAA	ARS606, ARS1305, ARS105, ARS112, ARS431, ARS520, ARS606, ARS733, ARS1305, ARS1330, ARS112, FRAII

CATGTCACCGACGCATCACCG	—

ATACACAGAAAATAGAAATGTCTTAA ATTTTTATATTTTTCACACTTTAAAGT	ARS215, ARS452, ARS440, ARS602, ARS110, ARS733, ARS920, ARS1021, ARS1127, ARS1211, ARS220, ARS215

AGGGCCCTAGAGGGGCCCTAG	—

**Table 10:** Curvature propensity and bendability of APS sequences.

S. number	APS	Curvature propensity	Position
1	ARS310	12.5	570
2	ARS315	12	40
3	ARS606	17.5	225
4	ARS1305	15	185
5	ARS1426	14.5	210
6	Ste20-YF1	10	650
7	Ste20-YF2	11.5	590
8	Ste20-YF3	11.75	580
9	Ste20-YF4	10	550
10	FSI	10	115, 410
11	FSII	15.5	200
