# Supplementary material for: Mechanism of Gene Amplification via Yeast Autonomously Replicating Sequences
Source: ScientificWorldJournal. 2015 Jan 20;2015:387367. doi: 10.1155/2015/387367 (PMC4320851; doi:10.1155/2015/387367)
Supplement: Supplementary file 1 — Combined analysis: To correlate the evolutionary trends of the ARS with their physical properties, a combined analysis of the eight parameters (i.e., free energy values (∆G), T REP values, origin efficiency, SIDD, flanking elements, co-localization of a possible fragile site, curvature and GC content) of each of these chromosomal ARS was conducted. This was compared with their closely placed members in the phylogenetic tree. A thorough comparison of the properties of the members of each cluster revealed interesting results. It was observed that in addition to the closeness in the phylogenetic tree, ARS 1316 and 1507 share similarity with respect to their physical properties too. They behave as confirmed origins of replication, have low curvature (9-10), low TREP and low G+C values. ARS 1620 which showed the longest branch length had high SIDD value, no flanking elements and low curvature (approximately 7). These properties were similar to the properties of ARS 701 which had the second longest branch length. ARS elements of chromosome IV were found to be located close to one another and approximately all the members showed similar properties, for example, ARS 409, 432, 418 showed replication efficiency, fragile site colocalization, high curvature, ∆G and T REP values (curvature 14–16 was noteworthy). Similarly, ARS 518, 503, 515, 519, 513 showed very similar properties. Also, ARS of chromosome IV and XII showed very similar properties, while ARS 308-1308 were more closely placed in the phylogram and this close relatedness was well reflected in their physical properties also (high ∆G, low curvature, presence of LTR and fragile sequences). [file 387367.f1.pdf]

Supplementary table I: Combined analysis of the *S. cerevisiae* ARS

Chr No.	ARS	G	TREP	OE	SIDD	FE	FS	RE	Curvature	G+C
**1**	102	89.06	0	0	0	tel	0	1	14	14
	103	75.14	27.5	1	3.33	0	3	0	10	10
	104	81.71	28.3	1	0.024	TY1,LTR	0	0	8	8
	105	76.47	34.1	1	0	0	2	1	13	14
	106	98.03	33.3	1	6.11	0	1	0	9	10
	107	77.4	29.9	1	-0.028	LTR	2	1	12	12
	108	79.56	18.2	1	0	0	5	1	12	12
	109/101	87.1	0	1	-0.13	RT, LTR	5	0		
	110	77.14	14.6	1	0	RT,LTR	0	0	10	11
	111	81.36	0	1	0	LTR	0	1	16	16
	112	79.36	29.9	1	0	0	4	1	14	16
	telo X	0	29.9	1	0	0	3			
**2**	230	81.87	0	0	4.49	LTR	2	1	12	12
	202	92.87	0	1	0	0	1	1	12	12
	207	88.85	0	1	0	0	1	0	10	10
	231	83.82	17.9	0	1.72	LTR	2	0	10	10

Continued…

	208	78.33	19.1	1	0	RT,LTR	2	0	9	9
	209	77.68	0	1	0	LTR	6	1	12	13
	211	88.17	0	1	0	LTR	0	0	11	12
	212	76.9	0	1	0	0	4			
	213	94.49	0	1	0	0	6			
	214	87.53	25.4	1	5.91	0	1	0	7	8
	215	86.58	25.7	1	5.65	0	0	1	14	16
	216	87.31	30.1	1	-0.031	0	1	0	10	11
	220	77.51	26.3	1	-0.66	0	2	0	10	12
	221	80.49	30.1	1	0.27	LTR	2			
	222	77.71	29.4	1	0.08	0	2	1	12	12
	224	85.98	30.3	1	8.3	0	2	0	10	10
	225	76.91	33.1	1	1.19	0	1	1	14	14
	228	77.01	37	1	0.37	0	1	0	9	9
**3**	300	78.61	0		0.94	LTR	0	0	8	8
	301	79.75	0		3.14	LTR	1	0	11	12
	302	0	0		4.47	0	2	1	14	14
	303	0	0		7.451	0	3	1	12	12
	320	77.64	0		0	0	5	1	14	14
	304	0	0		7.6	0	3	0	10	10

Continued…

	305	81.16	12.6		2	0	3	0	11	11
	306	83.2	10.8		-0.33	LTR	2	1	14	14
	307	73.47	19.8		3.33	LTR	3	0	10	10
	308	70.19	19.8		-0.4	LTR	3	0	10	10
	309	72.31	22.5		1.6	LTR	2	0	10	10
	310	78.43	24.9		0	LTR	4	1	12	14
	313	79.48	16.3		-0.28	0	2	0	10	12
	314	76.22	16.9		1.83	0	2	1	14	14
	314.5		16.9		0	0	0			
	315	88.34	14.8		1.19	0	1	1	13	14
	316	86.13	33.4		2.57	0	2	1	12	12
	317	62.83	31.1		-0.32	LTR	2	0	10	12
	318	67.72	31.1		4.83	LTR	2	1	12	14
	319	72.02	0		0	TEL	0	1	12	14
**4**	400	80.13	0		0	TEL	0	0	10	12
	403	0	0		0.65	0	0	0	9	9
	404	69.79	36.5		2.19	0	2	1	14	14
	405	70.44	34.2		-0.07	0	2	1	14	14
	405.5	0	0		0	0	0			
	406	71.36	29.4		-0.57	0	2	1	12	12

Continued…

	409	76.26	36		0.6	0	1	0	11	11
	409.5	0	33.2		-0.032	0	0			
	410	87.67	28.6		0.36	0	1	1	12	12
	412	82.66	0		2.5	0	0			
	413	62.79	20.5		-0.14	0	0	0	9	10
	413.5	0	0		0	0	0			
	414	83.01	26.3		-0.49	0	1	0	10	10
	415	80.83	19.9		-0.08	LTR	4			
	416	80.58	23.3		0.32	0	0			
	417	96.01	22		0.33	0	1	1	12	12
	417.5	0	24.1		6.19	0	0			
	450	89.61	0		0	LTR	3	1	12	12
	418	84.71	18		2.96	LTR	1	0	11	14
	419	83.75	0		1.32	0	2	1	14	12
	420	81.74	32.8		0.77	0	0			
	421	75.59	0		0.14	LTR	1	0	10	10
	422	82.28	32.8		0.93	0	2	0	10	10
	422.5	0	32.3		-0.67	0	0			
	451	0	0		0	0	1	0	9	9
	423	84	32.3		-0.77	0	2	0	11	11

Continued…

	425	92.94	38.8		7.04	LTR	1	0	9	9
	427	80.14	0		0	LTR	0			
	428	0	12		6.03	0	2	1	12	12
	429	81.61	12		0.01	LTR	2	0	10	10
	430	87.26	24.3		0.83	LTR	2	0	9	8
	430.5	0	0		0	0	0			
	431	77.74	32.6		-0.41	LTR	2	1	14	14
	431.5	0	35.9		-0.21	LTR	0			
	452	72.14	0		0	LTR	2	1	14	14
	432	86.34	12.6		0.02	0	1	0	10	10
	432.5	0	12.6		3.5	0	0			
	453	82.9	0		0	0	1	0	9	10
	433	88.65	36.7		1.88	0	2	0	11	11
	434	85.18	36.9		3.6	0	1	1	12	12
	435	73.92	32.9		-0.36	0	1	0	11	11
	439	75.42	0		0	0	0			
	440	85.96	30		-0.54	0	1	1	14	16
	441		29.4		0	0	0			
	443	76.54	0		0	0	2	0	11	10
	446	87.91	35.9		3.37	0	2	0	8	8

Continued...

**5**	502	51.71	0		8.7	0	0	0	4	4.5
	503	78.27	0		0	0	2	0	9	9
	504	82.76	0		0	0	4	1	16	16
	507	83.64	19.7		1.69	LTR	2	0	11	11
	508	81.2	14.3		1.98	LTR	1	0	10	10
	510	87.06	18.9		84.5	LTR	1	0	8	9
	511	80.46	18.7		2.37	0	1	1	12	12
	511.5	0	0		7.59	0	0			
	512	82	29.5		1.26	0	1	1	14	14
	513	78.33	0		0	LTR	3	0	10	10
	513.5	0	0		-0.31	LTR	0			
	514	91.04	22.6		6.85	LTR	1	1	12	12
	515	81.53	0		0	LTR	1	0	9	9
	516	82.47	24.3		1.76	LTR	1			
	517	89.58	25.3		5.01	0	1	1	12	12
	518	97.01	22.2		8.03	LTR	0	0	10	12
	519	90.7	22.2		6	LTR	1	1	12	14
	520	77.25	29.1		0	RT,LTR	4	1	16	16
	521	71.67	0		-0.26	LTR	2	0	9	10
	522	75.54	32.5		-0.41	LTR	1	0	10	12

Continued...

	523	79.23	34.4		0	LTR	2	0	10	12
**6**	600	82.31	0		0.32	0	0	0	11	11
	601	82.31	42.4		1.87	0	2	0	9	9
	602	83.15	42.4		0.4	0	1	1	12	14
	603	76.46	36.2		2.82	0	1	1	12	12
	603.1	0	0		-0.11	0	1			
	603.5	0	21.5		0.94	0	0	0	10	11
	604	91.84	21.5		8.42	0	0	1	12	12
	605	44.68	25.4		2.81	RT,LTR	0	0	5	5.5
	606	81.76	18.6		-0.39	LTR	1	1	18	18
	607	43.05	12.8		6.2	LTR	0	0	7	7
	608	67.73	0		-0.51	0	3	0	8	8
	608.5	0	0		-0.29	0	0			
	609	94.45	31.8		3.56	0	0	1	14	14
	610	78.53	0		0.31	TEL	3	0	10	12
**7**	702	80.82	32.6		7.28	0	2	0	10	11
	704	84.87	31.2		0.24	LTR	1	1	14	16
	706	82.96	31.2		-0.44	LTR	1	0	10	9
	707	75.84	31.4		-0.32	0	1	0	8	8

Continued…

	710	83.39	31.9		-0.03	LTR	1	1	16	16
	701/711	50.07	39.2		7.8	0	0			
	714	77.27	29.1		0.032	0	1	0	8	9
	716	84.69	33.9		2.58	0	1	0	8	8
	717	77.89	30.5		-0.51	LTR	1	0	8	9
	718	89.03	30.8		0.66	LTR	1	1	12	12
	719	88.03	24.7		4.57	0	1	0	9	10
	720	87.63	19.9		-0.32	0	1	0	6	6
	721	81.39	29.6		1.28	RT,LTR	2	0	8	8
	722	72.95	29.2		0.47	RT,LTR	0	0	10	10
	724	84.22	0		0	0	0			
	727	79.25	25.9		0.07	LTR	1	1	12	12
	728	76.23	30.6		-0.56	LTR	1	0	9	10
	729	92.95	24.9		5.59	RT,LTR	0	0	10	10
	731	83.66	22.5		3.34	0	1	0	9	9
	731.5/737	80.38	21.8		-0.55	0	1			
	735	81.17	0		0	0	0			
	733	0	34		-0.02	0	3	1	14	14
	734	80.67	0		0	0	0	1	12	11
	736	88.72	28.8		0	TEL	0			

Continued...

**8**	802	80.36	0		2.41	LTR	1	1	12	12
	801	78.55	0		0	0	0			
	805	0	26.4		1.83	RT,LTR	1	1	14	14
	806	86.02	0		0	LTR	0			
	807	83.81	23.4		5.24	LTR	2	0	9	10
	803	76.48	0		0	0	0			
	808	94.69	0		0	0	2	0	11	11
	809	78.41	32		4.11	0	1	0	7	7
	810	78.75	44.9		0.19	0	0			
	813	81.26	38.4		0.36	0	2	1	12	14
	815	74.34	25.8		-0.51	0	0			
	818	86.56	32.8		8.89	LTR	1	0	10	10
	820	79.75	25.4		-0.06	LTR	1	0	11	11
	822	84.13	34.9		4.07	LTR	1	0	8	8
	823	0	0		-0.37	0	0			
	824	73.85	0		1.86	TEL	0	1	12	14
**9**	907	0	29		0.14	0	2			
	902	77.02	0		0	0	0			
	903	0	0		0	0	2			
	904	73.16	0		0	0	3			

Continued...

	905	0	0		0	0	6			
	909	78.1	26.9		-0.02	0	1	0	10	10
	910	83.95	0		0	LTR	6			
	912	78.21	36.1		-0.49	0	2	1	14	14
	911.5	0	0		-0.39	0	1			
	901/913	0	24.7		-0.38	RT,LTR	1			
	913.5	0	0		1.53	LTR	1			
	923	93.77	0		0	0	0	0	10	10
	911	84.12	0		0	0	0	1	12	12
	914	93.53	0		0	0	2	0	10	10
	913	79.34	0		0	0	0	0	8	9
	915	88.55	0		0	0	7			
	916	0	36.9		-0.01	0	3			
	918	74.44	0		0	0	5			
	919	80.81	20.8		2.06	0	1	0	9	9
	920	73.5	20.6		4.21	0	1	1	14	14
	921	73.69	0		0	0	5			
	922	81.91	20.4		-0.21	LTR	1	0	10	9
	923	93.77	0		0	0	0			
**10**	1001	81.03	0		0.93	TEL	0	1	12	14

Continued...

	1002	80.37	0		9.09	0	1	1	14	14
	1003	73.16	0		-0.03	0	3	0	11	12
	1004	79.98	38.7		0.98	0	3	0	10	10
	1005	79.86	27		-0.32	LTR	2	0	11	12
	1006	0	24.8		-0.43	0	1	0	10	11
	1007	78	32.8		-0.34	0	3	0	9	9
	1007.5	0	26.5		3.45	0	2			
	1008	77.52	24.8		0	RT,LTR	2	0	11	11
	1009	78.71	25.6		0.89	0	3	0	10	11
	1009.5	0	0		5.39	0	3			
	1010	76.91	33.2		-0.12	0	3	1	14	14
	1011	85.19	31.4		4.98	0	1	1	12	12
	1012	84.97	15.6		8.58	LTR	1	0	7	7
	1013	72.41	15.6		-0.16	LTR	1	0	9	9
	1014	79.25	16.6		8.16	LTR	2	1	12	12
	1015	80.01	21.4		1.92	0	2	1	12	12
	1016	82.98	25.2		0	0	4	0	11	10
	1017	90	25.2		7.82	0	2	0	10	10
	1017.5	0	37.6		0	0	3			
	1018	84.47	17.3		4.38	LTR	1	0	10	10
	1018.5	0	19.1		3.58	0	3			

Continued…

	1019	93.37	19.1		0.07	0	4	1	12	12
	1020	92.37	24.4		7.42	0	1	1	14	14
	1021	87.69	16.1		3.78	0	2	1	14	14
	1022	81.84	31.7		3.29	0	1	1	12	12
	1023	80.77	31.7		0.64	0	0	0	10	10
	1024	86.5	35.9		8.18	0	1	1	12	12
	1025	78.63	35.9		7.44	0	0			
**11**	1104.5	0	26.2		-0.02	0	2			
	1103	73.73	23.4		4.43	LTR	2	0	9	9
	1104	80.1	0		0	LTR	6			
	1125	0	0		0	0	0	1	18	18
	1102	79.06	0		0	0	0			
	1106	73.06	26.3		0.47	LTR	1	1	14	14
	1126	77.86	0		0.27	0	2	1	11	12
	1127	88.87	27.4		2.21	LTR	1	1	14	14
	1109	73.06	25.5		-0.028	LTR	2	1	14	14
	1112	75.67	27.6		-0.36	0	2	1	12	14
	1113	73.28	23.89		-0.35	0	2	0	10	11
	1114	89.46	20.7		6.31	LTR	0	1	14	14
	1116	77.26	26.6		0.61	LTR	1	0	11	11

Continued...

	1118	83.05	29.6		0.77	0	1	0	8	9
	1120	82.41	30.1		0.94	0	1	1	12	12
	1123	85.79	32.6		2.2	LTR	1	0	9	9
	1125	79.31	0		0	0	0			
	1126	88.67	0		0	0	0			
**12**	1206	89.91	27.6		0.67	LTR	2	0	10	10
	1207	80.91	0		0	0	0			
	1208	80.5	22.9		-0.002	CEN	2	0	11	11
	1209	85.55	22.9		3.65	LTR	2	0	10	9
	1210	0	18.9		2.5	LTR	0			
	1211	89.73	18.9		6.77	LTR	1	1	14	14
	1211.5	0	20.9		0.12	0	4			
	1212	79.08	33.4		4.85	0	1	0	11	10
	1212.5	0	0		-0.65	0	1			
	1213	84.25	16.5		3.27	LTR	1	0	10	12
	1214	93.81	0		0	0	0			
	1215	85.29	21.4		3.89	0	0	0	10	9
	1216	83.98	24.8		8.26	LTR	0	0	9	9
	1216.5	0	0		7.47	0	0			
	1200-1	37.86	0		0	0	0	1	12	12

Continued…

	1200-2	88.33	0		0	0	0	1	12	12
	1217	82.96	21.8		-0.45	0	3	1	12	12
	1218	87.68	20		3.65	RT,LTR	1	0	11	11
	1219	87.68	0		0	0	0			
	1220	96.8	28.5		0.02	RT,LTR	1	1	12	12
	1223	83.1	18.5		3.38	0	1	0	10	10
	1226	87.064	30.2		7.35	LTR	2	0	8	8
	1238	87.13	35.5		-0.02	0	2	0	10	10
	1230	87.61	0		0	RT,LTR	0			
	1232	70.38	27.8		-0.22	0	1	1	12	12
	1233	78.91	27.8		-0.32	0	1	0	9	9
	1234	0	29.4		2.71	0	1	1	14	16
	1235	0	35.5		0.33	0	1			
**13**	1303	82.49	27.4		-0.46	0	1	1	14	16
	1304	81.77	27.4		-0.32	0	0			
	1305	78.24	22.9		-0.34	0	2	1	16	16
	1307	89.29	18.7		5.04	LTR	1	1	12	12
	1307.5	0	22.1		5.5	0	2			
	1308	83.34	12.3		8.87	RT,LTR	2	0	7	7
	1309	92.21	19.1		5.29	0	0	0	9	9

Continued…

	1310	89.61	14		6.14	LTR	1	1	14	14
	1312	83.34	23.2		3.06	RT,LTR	1	0	11	11
	1316	76.82	27.2		0.012	LTR	1	0	9	9
	1317	89.09	0		0	LTR	0			
	1320	70.84	20.4		2.54	0	2	0	9	9
	1319	80.19	18.8		1.1	LTR	2			
	1323	81.97	25.6		-0.28	0	1	1	12	12
	1322	83.5	0		-0.02	0	2			
	1324	83.83	0		-0.18	0	2	0	10	10
	1321	36.48	0		0	0	0			
	1325	80.19	20.4		1.45	0	2	1	12	12
	1327	90.65	27.6		2.43	0	2	1	16	16
	1328	92.95	0		6.21	LTR	1	1	12	12
	1329	71.77	0		-0.026	LTR	2	0	9	10
	1330	83.65	17.9		-0.38	LTR	1	1	14	14
	1331	87.3	0		0	LTR	0			
	1332	86.18	22.8		1.28	0	2	0	8	9
	1331.5	80.39	0		-0.48	0	2			
**14**	1405	85.12	32		2.9	0	1	0	9	9
	1406	81.23	27.8		-0.17	0	2	1	12	12

Continued…

	1407	83.4	25.5		2.23	0	1	0	10	12
	1409	0	0		0	RT,LTR	0	0	5	5
	1410	99.42	0		0	0	0	0	9	9
	1411	89.66	30.4		8.57	0	1	0	7	7
	1412	0	29.6		0.16	0	2	0	10	11
	1413	78.29	29.1		1.68	0	1	0	10	12
	1414	73.6	24.7		-0.28	0	2	0	11	11
	1415	82.77	21		0.33	0	4	1	14	14
	1416	80.71	27.1		6.93	0	0	0	9	9
	1417	86.17	0		0	0	0	1	14	14
	1419	83.9	29.5		1.26	0	2	1	14	14
	1420	82.91	27.2		5.36	0	1		12	12
	1421	88.78	0		2.14	LTR	2	0	10	9
	1422	79.64	15.7		-0.48	LTR	2	0	9	9
	1423	77.73	20.7		-0.15	RT,LTR	5	0	8	8
	1424	72.42	20.7		0	0	2	0	11	11
	1425	84.97	0		0	0	0	0	10	10
	1426	74.85	24.7		-0.35	LTR	1	1	15	15
	1427	74.85	24.1		4.79	0	1	0	10	10
**15**	1506.5	0	24.5		0.12	0	0			

Continued…

	1531	0	0		0	0	4	1	12	13
	1501	77.54	0		0	0	0	0	9	9
	1507	0	0		-0.13	0	0	0	10	10
	1502	43.93	0		0	0	0	0	6.7	6.7
	1508	82.03	20.6		0.43	0	2	0	8	9
	1509	80.48	18.8		-0.09	RT.LTR	1	0	10	10
	1509.5	0	0		-0.04	0	4			
	1510	85.1	14.1		4.04	0	2	0	10	12
	1510.5	0	17.2		4.83	0	0			
	1511	81.65	12.4		-0.36	0	0	0	8	9
	1512	94.54	0		0	LTR	6	1	12	10
	1513	76.5	17.6		-0.07	LTR	1	1	12	12
	1513.5	77.54	19		-0.31	LTR	1			
	1514	0	19.1		-0.29	0	2			
	1516	0	22		-0.13	0	1			
	1517	0	22.14		-0.18	0	0			
	1518	88.26	0		0	RT,LTR	7	0	9	9
	1519	86.55	24.5		-0.18	LTR	2	1	12	12
	1521	0	0		-0.22	RT,LTR	1	0	11	11
	1523	72.88	25.9		-0.47	0	1	1	12	12

Continued...

	1524	91.2	26.9		-0.01	0	1	0	10	10
	1526	86.4	21.3		2.85	0	2	0	7	8
	1528	81.18	0		0	0	1	0	10	10
	1529.5	0	18.6		-0.61	0	0			
	1529	90.43	26.7		6.1	RT,LTR	2	1	13	13
**16**	1601	0	29.9		0	0	2			
	1602	43.93	29.9		0	RT,LTR	0			
	1600	80.43	0		0	0	0			
	1603	94.96	0		0	0	0			
	1604	74.456	29.2		-0.42	RT,LTR	3	1	13	14
	1605	88.04	22.1		2.6	RT,LTR	1	0	7	8
	1607	83.45	26.4		1.44	0	2	0	10	10
	1611	41.07	0		0	LTR	0			
	1614	78.81	20.6		-0.5	0	1	1	12	12
	1608	67.73	0		0	0	0			
	1618	84.9	20.8		0.81	0	1	0	11	11
	1618.5	0	16.5		0	0	8			
	1619	87.5	16.5		0.68	0	2	0	10	10
	1633/1620	0	24.2		2.15	0	0			
	1621	80.72	22.3		1.4	RT,LTR	1			

Continued…

	1622	88.34	21.5		2.96	LTR	2	0	9	10
	1622.5	0	21.5		-0.51	LTR	1			
	1623	87.54	19.3		1.12	0	2	0	9	9
	1624	70.55	22.7		-0.34	0	2	0	11	11
	1625	93.01	22.7		6.13	0	1	1	14	14
	1626	74.85	0		-0.32	0	2	1	12	12
	1626.5	0	15.9		0.56	0	0			
	1635	0	0		0	LTR	1	1	12	12
	1627	80.92	18.7		0.36	0	1	1	12	12
	1628	67.66	21.7		-0.37	RT,LTR	2	1	14	14
	1629	43.92	0		0	RT,LTR	0			
	1630	76.12	28.7		3.39	0	2	0	10	10
	1631	83.28	22.1		4.61	0	0	0	7	7
	1632	0	0		0	0	0			
	1633	19.46	0		0	0	0	1	14	14
	1620.5	0	0		0	0	2			

*Blank coloumns indicate that respective ARS failed to yield values for that parameter
